# Anaplastic Lymphoma Kinase (ALK) Mutation-Targeting Treatment With Alectinib in Lung Adenocarcinoma and Primary Cutaneous Marginal Zone B-Cell Lymphoma

**DOI:** 10.7759/cureus.29922

**Published:** 2022-10-04

**Authors:** Sethi Ashish, Robin R Rodriguez, Purva Sethi, Fang Yu, Moses Raj

**Affiliations:** 1 Medical Oncology, Allegheny Health Network, Pittsburgh, USA; 2 Oncology, Allegheny Health Network, Pittsburgh, USA; 3 Research, Butler Health System, Butler, USA; 4 Medical Oncology, University of Pittsburgh, Pittsburgh, USA; 5 Hematology & Oncology, Allegheny Health Network, Pittsburgh, USA

**Keywords:** central nervous system metastasis, adaura trials, primary cutaneous b-cell lymphoma, acletinib, targeted therapy, tyrosine kinase inhibitors (tki), non-small-cell lung carcinoma (nsclc), alk mutation, alk inhibitor

## Abstract

Lung adenocarcinoma or non-small cell lung cancer (NSCLC) represents one of the most diagnosed cancers worldwide. Anaplastic lymphoma kinase (ALK) mutation, a tyrosine kinase and ALK fusion or rearrangement oncogene, has been found rarely in patients with NSCLC. Newer treatment modalities with different ALK inhibitors in targetable specific ALK mutations have recently made great strides in the management of NSCLC patients. We present a case of NSCLC harboring ALK mutation with primary cutaneous marginal zone B-cell lymphoma (PCMZL) treated with adjuvant chemotherapy with pemetrexed and cisplatin, and ALK-echinoderm microtubule-associated protein-like 4 (EML4)-targeting treatment alectinib.

## Introduction

Anaplastic lymphoma kinase (ALK) is a tyrosine kinase expressed in approximately 3%-5% of NSCLC tumors [1-2] and is usually seen in younger patients who have no smoking history. We present a case of NSCLC harboring ALK mutation with primary cutaneous marginal zone B-cell lymphoma (PCMZL) treated with adjuvant chemotherapy with Pemetrexed-Cisplatin combination and ALK-echinoderm microtubule-associated protein-like 4 (EML4)-targeting treatment alectinib. The patient has been in remission since one year after the treatment with alectinib at the time of reporting this case report. Surprisingly rash was also resolved before starting alectinib when the patient was on pemetrexed and cisplatin for NSCLC in the adjuvant setting.

## Case presentation

**Figure 1:**
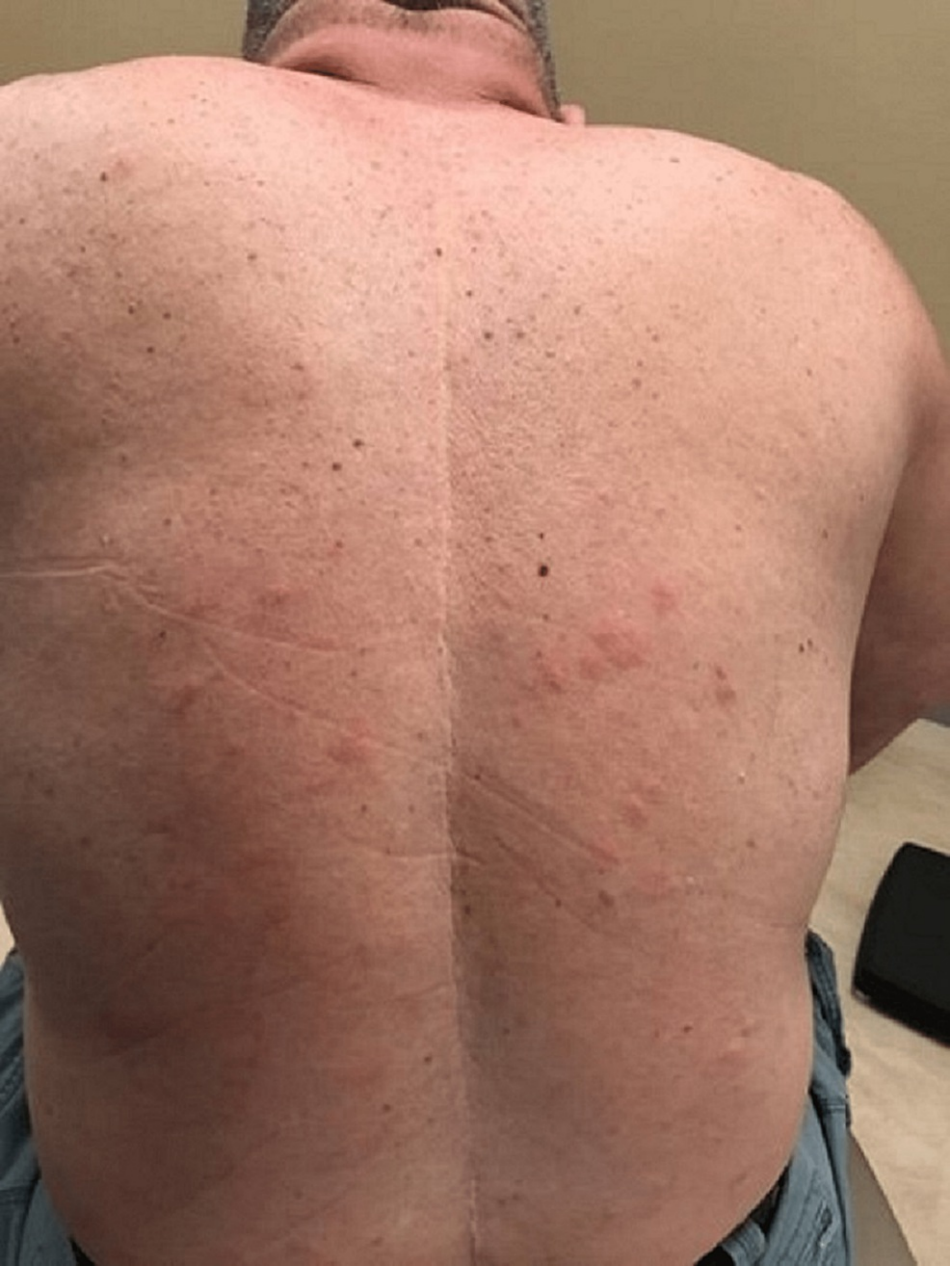
Rash: primary cutaneous marginal zone B-cell lymphoma. Plaques seen PCMZL, primary cutaneous marginal zone B-cell lymphoma

**Figure 2:**
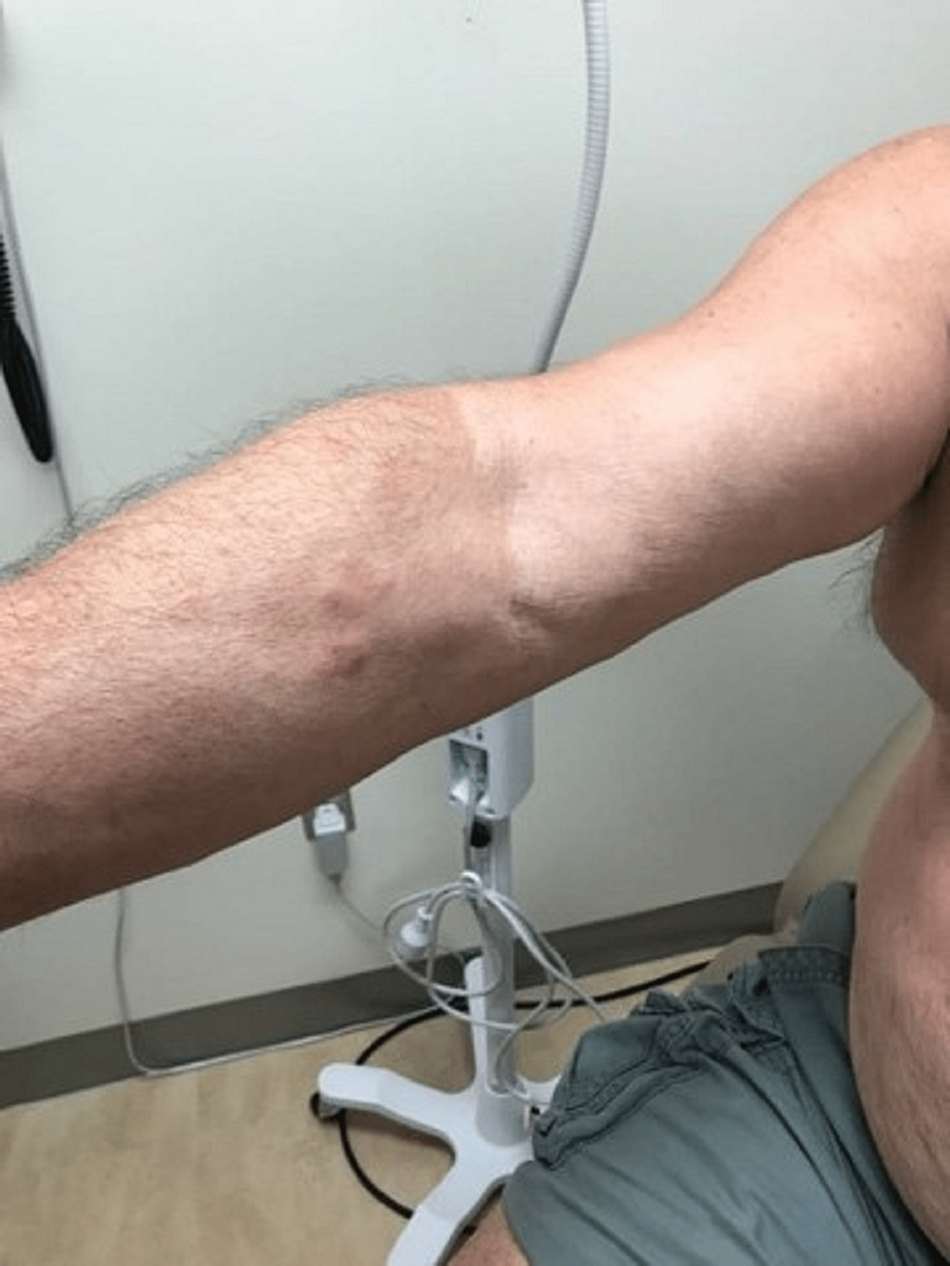
Primary cutaneous marginal zone B-cell lymphoma. Right forearm: plaques

**Figure 3:**
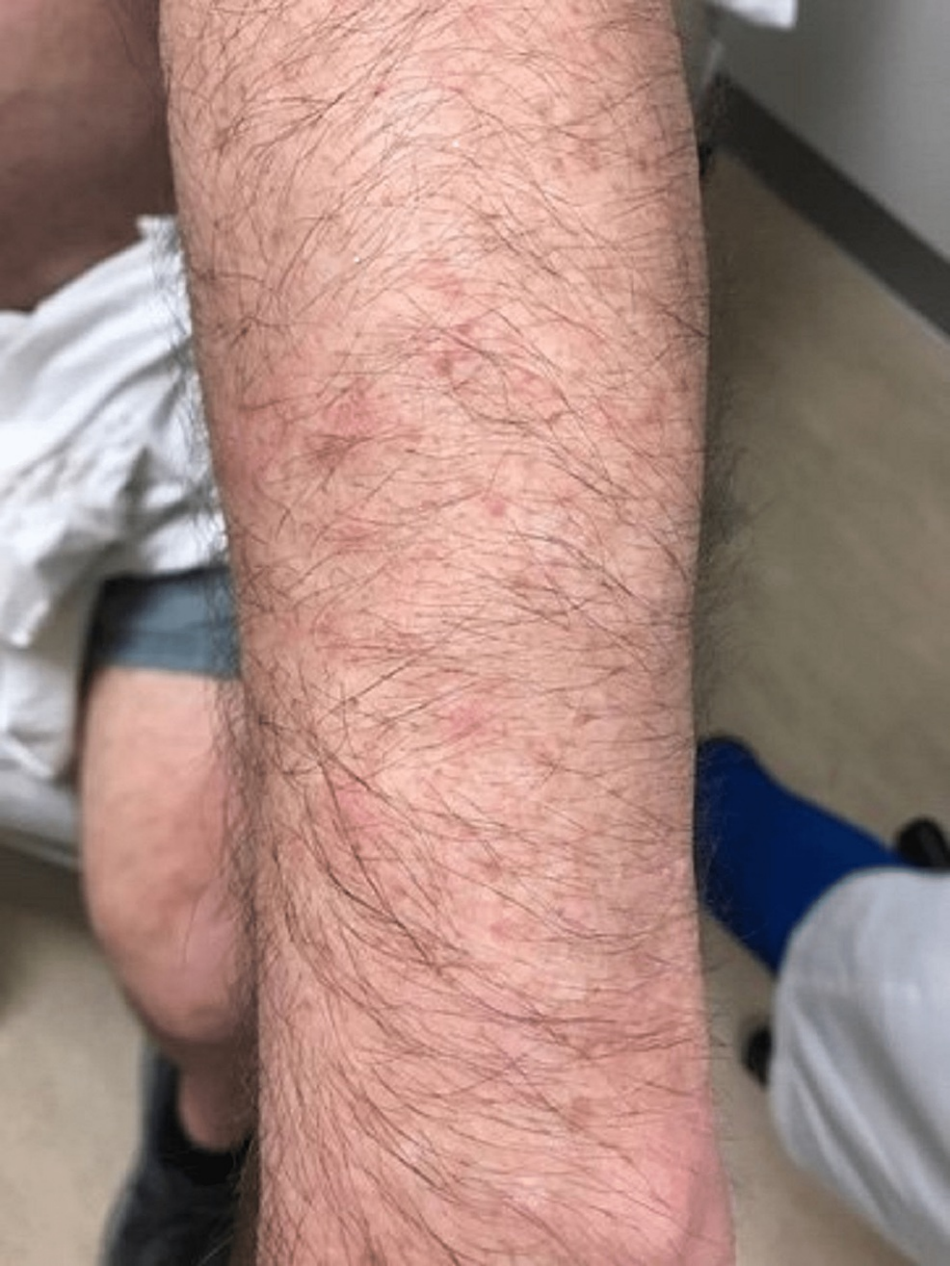
Primary cutaneous marginal zone B-cell lymphoma. Right forearm plaques

**Figure 4:**
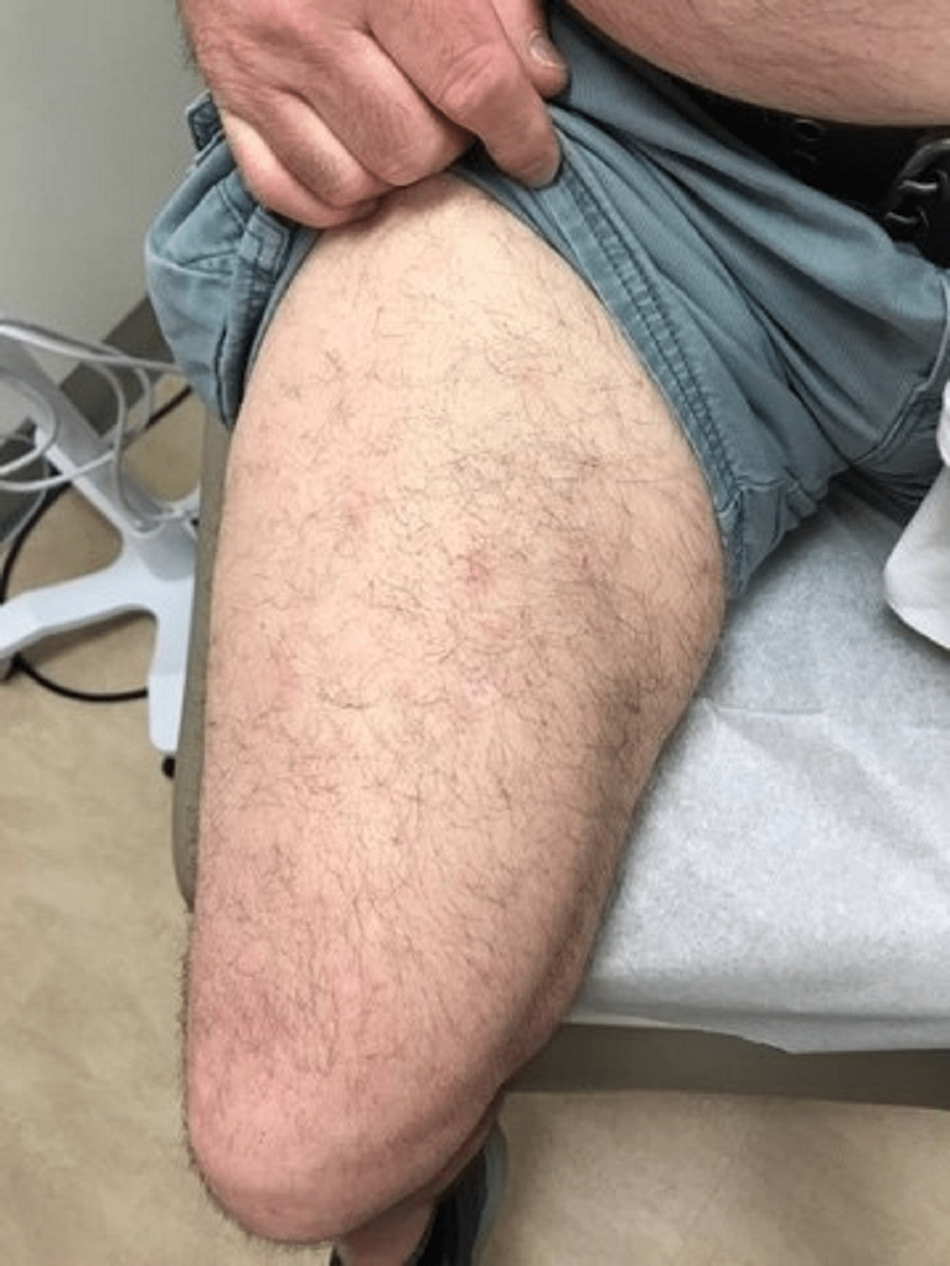
Primary cutaneous marginal zone B-cell lymphoma. Right thigh: plaques

A case of a 68-year-old Caucasian male with no smoking history was initially seen by dermatology for a chief complaint of rash mostly located on the arms, legs, and the back (Figures 1-4). The rash had been present for more than two years and was itchy, red, and moderate in severity, which made the patient seek care.

The dermatologist performed a biopsy from one of the bumpy-looking lesions seen from the left shoulder and confirmed a diagnosis of PCMZL, phenotyped as CD20+, CD30-, bcl2+, bcl6+/-, and Ki67 at 10%. However, the patient did not show any concerning features or B symptoms of systemic lymphoma such as fever, night sweats, weight loss, or lymphadenopathy. During workup tests for PCMZL, a positron emission tomography (PET) and a CT were done that showed a mildly hypermetabolic sub-centimeter nodule in the right lung and significantly hypermetabolic right hilar and right paratracheal lymph nodes (LNs) (right hilar LN measured approximately 2.6 cm x 1.6 cm and the largest right paratracheal LN measured 1.4 cm x 1.3 cm). LNs were then sampled and biopsied, which confirmed levels 4R,2R,10R, and 7 positive for primary pulmonary adenocarcinoma. The patient then underwent bronchoscopy, right middle lobectomy, and right upper lobe wedge resection, and was diagnosed as stage III (T2aN2M0) right lung adenocarcinoma. The surgical biopsy also revealed 10 out of 25 LNs involved in staged cancer. Tumor immunohistochemical (IHC) profile showed positive results for TTF-1 (thyroid transcription factor-1), cytokeratin (CK)7, AE1/3, but were negative for CK20, GATA-3, PAX-8, CDX-2, or NKX3.1. Foundation one companion diagnostic (CDx) on lung specimen presented ALK rearrangement intron 19 and fusion with EML4. Programmed death ligand-1 (PDL1) was positive at 1%. Due to low tumor purity, microsatellite status and tumor mutational burden (TMB) were not able to be determined. Treatment of lung adenocarcinoma took precedence over the treatment of PCMZL. Although his disease was extensive in the skin there was no evidence or clinical presentation of any systemic disease. First-line adjuvant chemotherapy with pemetrexed and cisplatin was initiated one month after the surgery, while alectinib for targeting ALK-EML4 gene mutation was initiated at the completion of three cycles of adjunctive chemotherapy. The patient had high-risk features of ALK mutation NSCLC with 10 out of 25 LNs positive which carries more potential for central nervous system (CNS) metastasis. Treating with alectinib in the adjuvant setting with such high-risk features can have the potential of increasing the disease-free survival (DFS).

Following the initiation of adjuvant chemotherapy with pemetrexed and cisplatin, and ALK-EML4-targeting treatment alectinib, the patient tolerated the regimen very well, experiencing only mild fatigue with consistent dry cough. The PCMZL skin nodules were also significantly improved after the completion of four cycles of pemetrexed and cisplatin. A comprehensive CT scan was negative for any cancer recurrence findings. The patient has been in remission since one year after the treatment with alectinib at the time of reporting of this case report.

## Discussion

Fusion of the ALK-EML4 genes in NSCLC can be detected in tissue samples by means of various methods such as fluorescence in situ hybridization (FISH), quantitative reverse transcriptase polymerase chain reaction (qRT-PCT), and immunohistochemistry (IHC) [3]. Several generations of oral inhibitors targeting ALK fusion-positive mutation have entered the market and benefited ALK fusion NSCLC patients by improving survival and decreasing CNS metastasis (Table 1). Alectinib has improved ALK inhibition and blood-brain barrier transport when compared with crizotinib and reports concluded longer median disease-free survival [4-6] as well as an improved safety profile in NSCLC compared with crizotinib. While most patients tolerate the treatment well, resistance and side effects such as nausea, vomiting, diarrhea, constipation, change of vision and pneumonitis, lend to the need for precautions while treating NSCLC patients with ALK mutation. EML4-ALK mutation may be linked with increased PD-L1 expression [7]. Accordingly, immune checkpoint inhibitors (ICIs) are expected to have efficacy in the treatment of NSCLC with ALK rearrangements. However, there is increased toxicity, and should not be used with ALK inhibitors. CheckMate 370 addressed the safety of nivolumab (240 mg once every two weeks) with crizotinib (250 mg twice daily) as initial treatment for previously untreated ALK-rearranged advanced NSCLC patients [8]. Out of 13 patients, five (38%) developed hepatotoxicity and discontinued the combination therapy, with two patients dying. The overall response rate (ORR) was reported as 38% [8]. 

**Table 1:** ALK inhibitors. ROS1, ROS proto-oncogene 1, receptor tyrosine kinase; EGFR, epidermal growth factor receptor; TKIs, tyrosine kinase inhibitors; CNS, central nervous system; ALK, anaplastic lymphoma kinase

ALK Inhibitors	Special characteristics
Crizotinib (1^st^ generation) TKI	The resistance of crizotinib develops inevitably after ten months of therapy initiation
Alectinib (2^nd^ generation) TKI	Higher CNS activities and systemic efficacy provides better intracranial disease control and remains effective in those failing response crizotinib due to resistance
Brigatinib (2^nd^ generation) TKI	Targets broader ranges of ALK mutations and ROS1 rearrangements. The only ALK inhibitor which also targets EGFR
Ceritinib (2^nd^ generation) TKI	Higher potency than crizotinib
Loratinib (3^rd^ generation) TKI	Also targets ROS proto-oncogene 1, receptor tyrosine kinase ROS1

The PROFILE 1007 study reported pemetrexed as salvage therapy has efficacy in ALK-rearranged NSCLC, with a median progression-free survival (PFS) of 4.2 months in the pemetrexed arm versus 2.6 months in the docetaxel arm, indicating pemetrexed being more effective [9]. However, resistance against ALK-TKIs remains a clinical concern.

Further study regarding the adjuvant use of targeted therapy among those with targetable genetic alterations is ongoing. The ALCHEMIST trial is actively enrolling patients with resectable NSCLC and will perform genetic testing of their tumors [10]. Patients with tumors that have classic epidermal growth factor receptor (EGFR) mutations were offered adjuvant erlotinib versus observation, and this study has now been completed [10]. Patients with ALK gene rearrangement in their tumor are offered adjuvant crizotinib or observation [10]. Crizotinib is superior in patients with NSCLC after progression following platinum-based chemotherapy [11]. Similarly, the DFS benefit in ADAURA trial with osimertinib versus placebo in disease stages IB to IIIA NSCLC, with or without previous chemotherapy [12], suggests that adjuvant osimertinib could be an effective treatment option for patients, regardless of adjuvant chemotherapy use. Therefore, based on this analysis we advocate the need for EGFR mutation testing in all NSCLC disease stages, not only in advanced disease [12], for treatment decisions that can effectively be implemented in cases of ALK mutation positive patients as well. 

## Conclusions

The pathogenesis and most effective treatment approaches for NSCLC with different lymphomas have yet to be elucidated. Our patient presented with ALK mutation in NSCLC and PCMZL rash which resolved after pemetrexed and cisplatin combination. Alectinib or TKIs and chemotherapy combination is an uncommon phenomenon. Also combining immunotherapy with TKIs may result in many side effects and is not a useful combination treatment. Although pemetrexed is reported to have efficacy in cases with ALK-EML4 translocation, this finding has not been replicated in cases of dual altered lung cancer and lymphoma. Thus, any patients harboring driver mutation with high-risk lung cancers features can improve survival if treated with targeted therapy in the adjuvant setting.
